# High levels of pretreatment and acquired HIV drug resistance in Nicaragua: results from the first nationally representative survey, 2016

**DOI:** 10.1002/jia2.25429

**Published:** 2019-12-20

**Authors:** Amalia Girón‐Callejas, Claudia García‐Morales, Ricardo Mendizabal‐Burastero, Matilde Román, Daniela Tapia‐Trejo, Marissa Pérez‐García, Verónica S Quiroz‐Morales, Sandra I Juárez, Giovanni Ravasi, Carlos Vargas, René Gutiérrez, Luz Romero, Aleyda Solórzano, Edgar Sajquim, Sanny Northbrook, Santiago Ávila‐Ríos, Gustavo Reyes‐Terán

**Affiliations:** ^1^ Universidad del Valle de Guatemala Guatemala City Guatemala; ^2^ Centre for Research in Infectious Diseases National Institute of Respiratory Diseases Mexico City Mexico; ^3^ Ministry of Health of Nicaragua Managua Nicaragua; ^4^ U.S. Centers for Disease Control and Prevention Guatemala City Guatemala; ^5^ Pan‐American Health Organization Washington DC USA; ^6^ Universidad del Valle de Guatemala Managua Nicaragua

**Keywords:** HIV, drug resistance, Nicaragua, World Health Organization, antiretroviral therapy, treatment failure, surveillance

## Abstract

**Introduction:**

A nationally representative HIV drug resistance (HIVDR) survey in Nicaragua was conducted to estimate the prevalence of pretreatment HIVDR (PDR) among antiretroviral therapy (ART) initiators and acquired HIVDR among people living with HIV (PLHIV) who had received ART for 12 ± 3 months (ADR12) and ≥48 months (ADR48).

**Methods:**

A nationwide cross‐sectional survey with a two‐stage cluster sampling was conducted from March to November 2016. Nineteen of 45 total ART clinics representing >90% of the national cohort of adults on ART were included. ART initiators were defined as PLHIV initiating or reinitiating first‐line ART. HIVDR was assessed for protease, reverse transcriptase and integrase Sanger sequences using the Stanford HIVdb algorithm. Viral load (VL) suppression was defined as <1000 copies/mL. Results were weighted according to the survey design.

**Results and discussion:**

A total of 638 participants were enrolled (PDR: 171; ADR12: 114; ADR48: 353). The proportion of ART initiators with prior exposure to antiretrovirals (ARVs) was 12.3% (95% CI: 5.8% to 24.3%). PDR prevalence to any drug was 23.4% (95% CI: 14.4% to 35.6%), and 19.3% (95% CI: 12.2% to 29.1%) to non‐nucleoside reverse transcriptase inhibitors (NNRTI). NNRTI PDR was higher in ART initiators with previous ARV exposure compared with those with no exposure (76.2% vs. 11.0%, *p* < 0.001). Protease inhibitors (PI) and integrase strand transfer inhibitors PDR was not observed. VL suppression rate was 77.8% (95% CI: 67.1% to 85.8%) in ADR12 and 70.3% (95% CI: 66.7% to 73.8%) in ADR48. ADR12 prevalence to any drug among PLHIV without VL suppression was 85.1% (95% CI: 66.1% to 94.4%), 82.4% to NNRTI and 70.2% to nucleoside reverse transcriptase inhibitors (NRTI). ADR48 prevalence to any drug among PLHIV without VL suppression was 75.5% (95% CI: 63.5% to 84.5 %), 70.7% to NNRTI, 59.4% to NRTI and 4.6% to PI.

**Conclusions:**

Despite implementation challenges yielding low‐precision HIVDR estimates, high rates of NNRTI PDR were observed in Nicaragua, suggesting consideration of non‐NNRTI‐based first‐line regimens for ART initiators. Strengthened HIVDR monitoring, systematic VL testing, and improved ART adherence support are also warranted.

## 1 Introduction

A recent meta‐analysis showed increasing trend in HIV drug resistance (DR) to non‐nucleoside reverse transcriptase inhibitors (NNRTI) in people living with HIV (PLHIV) starting first‐line antiretroviral therapy (ART) in Latin America since 2007 1. This emergence of NNRTI pretreatment drug resistance (PDR) threatens the effectiveness of first‐line NNRTI‐based ART, currently the preferred option in most countries of the region 2.

In Nicaragua, ART scale‐up has been ongoing since 2003 3. In 2018, among 9400 estimated PLHIV, 5000 (53%) were on ART and 3,700 (40%) had achieved viral load (VL) suppression 4. ART abandonment rates as high as 30% have been previously reported 5. Low VL suppression rate represents an important risk for acquired drug resistance (ADR) and further PDR transmission 6. According to the national ART guidelines, the preferred first‐line regimen for adults in Nicaragua is NNRTI‐based (efavirenz (EFV)) and a protease inhibitor (PI)‐based second‐line regimen is used after confirmed viral failure (two consecutive unsuppressed VL measurements within a 2‐month interval with adherence support, after at least six months on ART) 3. VL and CD4 count are performed semi‐annually for ART monitoring, but HIVDR testing is not routinely available 3. A 2011 to 2015 sentinel survey among ART‐naive PLHIV enrolled at Nicaragua's largest HIV clinic, Roberto Calderon Hospital, found increasing rates of NNRTI PDR, with overall prevalence of 11.3% (95% confidence interval (CI): 7.9% to 15.6%) 7.

In response to rising levels of NNRTI PDR in low‐ and middle‐income countries (LMIC), the World Health Organization (WHO) developed a Global Action Plan with a 5‐year framework 8 that strongly recommends the implementation of nationally representative HIVDR surveys based on a standardized methodology 9. Implementation of HIVDR surveillance strategies at the country level is critical to not only inform policy and advocate for updated ART guidelines, but also to better understand HIVDR locally 6, 10.

Given the worrying scenario of HIVDR in Nicaragua and following WHO recommendations, we performed a nationally representative survey to estimate PDR and ADR among PLHIV on ART for 12 ± 3 months (ADR12) and ≥48 months (ADR48).

## 2 Methods

### 2.1 Study design

We carried out a cross‐sectional survey with a two‐stage cluster sampling, following WHO‐recommended methods 11, 12. Nineteen out of a total of 45 ART clinics were selected, excluding smaller clinics (n = 26) that combined comprised <10% of the national cohort of adults on ART. The sample size was calculated based on the probability proportional to proxy size sampling approach to obtain outcome estimates with a CI of ±5%. The following model assumptions were made for PDR survey sample size calculation: 10% prevalence of PDR, 20% genotyping failure, 25% of ART initiators with prior exposure to ARVs, and 100% expected proportion of individuals initiating ART with NNRTI‐based regimens; and for ADR surveys sample size calculation: 85% expected prevalence of VL suppression, 15% expected laboratory (VL and genotyping) failure rate, 95% expected proportion of persons still on first‐line ART, and 100% expected proportion of individuals on first‐line NNRTI‐based regimens. The total sample size per survey was calculated using the WHO standardized Microsoft Excel‐based calculator 13, and the number of participants per clinic were assigned proportionally to clinic size. Target sample sizes were 239 for PDR, 262 for ADR12 and 376 for ADR48.

### 2.2 Survey enrolment

ART initiators ≥18 years, defined as PLHIV initiating (self‐reporting no prior exposure to ARV and including persons with unknown exposure to ARVs) or reinitiating (after experiencing an ART interruption of at least three months) first‐line ART, were included in the PDR survey. PLHIV (≥18 years old) on ART for 12 (±3) months for ADR12 or ≥48 months for ADR48, were included. Participant enrolment took place during March–November 2016. All eligible PLHIV were approached and those who voluntarily consented to participate in the survey were included until the sample size at each clinic was reached or until the end of the enrolment period.

Demographic data were collected on paper‐based forms by trained Ministry of Health staff, using a survey identification code linked to VL, CD4 count and HIVDR testing results.

### 2.3 Specimen collection, handling, storage and laboratory procedures

Whole blood specimens were collected and shipped to the WHO‐designated regional reference laboratory in Mexico City for VL, CD4 count and HIVDR testing within a week from sample collection, following laboratory standard operational procedures.

HIVDR testing was performed on specimens with VL ≥1000 copies/mL according to the WHO/HIV ResNet Laboratory Operational Framework 14. Briefly, HIV protease and reverse transcriptase were amplified from plasma virus, using an in‐house‐validated protocol (HXB2: 2243 to 3304) and sequences were obtained on a 3730xl Genetic Analyser (ThermoFisher, Waltham, MA) 15. Integrase (IN) was amplified and sequenced separately, using an in‐house‐validated protocol (HXB2: 4013 to 5265) 16, 17.

Sequences were assembled using the web‐based automated sequence analysis tool ReCall (British Columbia Centre for Excellence in HIV/AIDS, Vancouver, Canada) 18. Post‐testing quality assurance was carried out using the WHO HIVDR quality control tool (http://pssm.cfenet.ubc.ca/who_qc/). All sequence pairs with genetic distance <0.5% within the same sequencing batch were repeated and confirmed.

### 2.4 Data analysis

VL suppression was defined as <1000 copies/mL. Any HIVDR was defined as the presence of a penalty score ≥15 using the Stanford HIVdb tool (v8.2) 19 to one or more of the following ARVs: nevirapine (NVP), EFV, any nucleoside reverse transcriptase inhibitor (NRTI), boosted darunavir, boosted lopinavir (LPV/r) or boosted atazanavir. HIVDR to NNRTI referred to NVP, EFV or both. HIVDR to the preferred first‐line ART regimen referred to tenofovir (TDF) + emtricitabine (FTC) + EFV. HIVDR to the preferred second‐line ART regimen referred to zidovudine (AZT) + lamivudine (3TC) + LPV/r. HIV subtype was assigned using REGA HIV‐1 subtyping tool V3 20.

Data analysis was performed following the HIVDR WHO Data Analysis guidelines using STATA 15.1 (StataCorp, College Station, TX, USA), and accounted for all‐sites survey design and sampling weights 11, 12. The outputs were weighted considering the clinic sampling weight, the estimated eligible population sizes per clinic, the number of eligible individuals enrolled and the number of sequences available.

Retention‐adjusted VL suppression was calculated using the country estimate of adults known to be on ART 12 months after ART initiation (72.0%) 21 multiplied by the VL suppression estimates, and a Wald 95% CI was calculated using the associated variance.

### 2.5 Ethics statement

The institutional review boards of the Universidad del Valle de Guatemala and National Institute of Respiratory Diseases in Mexico revised and approved the study. All participants provided written informed consent prior to enrolment.

## 3 Results and discussion

### 3.1 Clinical and demographic characteristics of participants

A total of 638 participants were enrolled in the surveys: 171 (71.5% of the sampling goal) for PDR, 114 (43.5%) for ADR12 and 353 (93.8%) for ADR48. Among ART initiators, 71.9% (123/171) were male, 43.8% (74/171) had CD4 count <200 cells/µL, and 86.0% (148/171) initiated ART with the preferred first‐line ART regimen (TDF + FTC+EFV). Among PLHIV on ART, 97.8% were on first‐line at 12 months of ART initiation, decreasing to 84.8% at ≥48 months on ART (Table 1).

**Table 1:** Demographic and clinical characteristics of ART initiators and PLHIV on ART in Nicaragua, 2016^a^

	ART initiators (PDR; N = 171)			PLHIV on ART					
				12 ± 3 months (ADR12; N = 114)			≥48 months (ADR48; N = 353)		
	n	median or %	IQR or 95% CI	n	median or %	IQR or 95% CI	n	median or %	IQR or 95% CI
Sex									
Male	123	71.9	64.3 to 78.5	76	66.6	56.0 to 75.7	219	57.0	49.5 to 64.2
Female	48	28.1	21.5 to 35.7	38	33.5	24.3 to 44.0	134	43.0	35.8 to 50.5
Age (years), median (IQR)		32	26 to 42		32	26 to 39		37.0	32 to 48
≤25	41	24.6	20.5 to 29.1	27	33.3	23.9 to 44.4	27	9.0	5.7 to 14.0
>25	30	75.4	70.9 to 79.5	87	66.7	55.6 to 76.1	326	91.0	86.0 to 94.3
CD4 count (cells/µL), median (IQR)		247	82 to 432		400	213 to 620		416	202 to 654
<200	74	43.8	33.9 to 54.2	25	17.2	13.2 to 22.1	87	23.7	20.1 to 27.7
200 to 500	66	38.3	32.1 to 45.0	41	40.1	29.4 to 51.9	130	36.4	32.1 to 41.0
>500	31	17.9	9.9 to 30.2	48	42.7	31.8 to 54.3	136	39.9	35.3 to 44.6
ART regimen									
First‐line^b^	70	99.4	94.9 to 99.9	10	97.8	97.7 to 97.9	303	84.8	79.2 to 89.1
Preferred first‐line^c^	48	85.9	76.7 to 91.8	91	75.2	64.7 to 83.3	17	32.4	26.8 to 38.6
Second‐line	NA			4	2.2	2.1 to 2.3	49	14.4	10.4 to 19.5
Third‐line	NA			NA			1	0.8	0.1 to 5.6
No data	1	0.6	0.0 to 5.0	NA			NA		

ART, antiretroviral therapy; CI, confidence interval; EFV, efavirenz; FTC, emtricitabine; IQR, interquartile range; NA, not applicable; TDF, tenofovir.

a Study design‐weighted proportions and 95% confidence interval

b one ART initiator had missing data for initiated first‐line ART

c preferred first‐line ART regimen: TDF + FTC + EFV.

The most important limitation of the present study is that target sample size for the PDR and ADR12 surveys was not achieved due to logistic constrains, low national ART retention rate and short enrolment periods for some clinics. This is a critical limitation that may impact representativeness of the surveys due to enrolment bias, and affects precision of the survey estimates. To account for this limitation, survey outputs were weighted considering the eligible population size per clinic, the number of eligible individuals enrolled and the number of successful genotypes. Nevertheless, the demographic characteristics of the study population, including female‐to‐male ratio, age and geographical distribution were similar to those reported for the Nicaraguan population living with HIV 4, 5, 22, 23. A second important limitation is that differences between the actual data and some model assumptions for sample size calculations exist, including the expected PDR prevalence, the proportion of initiators with prior ARV exposure and the VL suppression rate for ADR surveys. This could also affect precision of the outcomes with wider CIs, especially for subgroup estimates.

### 3.2 Pretreatment HIV drug resistance survey

The weighted national estimate of ART initiators with prior exposure to ARVs was 12.3% (95% CI: 5.8% to 24.3%) and 85.4% (75.4% to 91.7%) for ART‐naïve. 2.3% (0.1% to 5.4%) had unknown prior exposure to ARVs. Of the 21 ART initiators reporting prior exposure to ARVs, eight reported that it was from taking ARVs for prevention of mother‐to‐child transmission of HIV (PMTCT), two from prior ART, one from pre‐exposure prophylaxis and ten were not able to identify the type of prior ARV exposure. Four ART initiators had missing data for prior ARV exposure history.

Most ART initiators with genotype results (168/171, 98.2%, 95% CI: 92.5 to 99.6) were infected with HIV‐1 subtype B, 0.6% (1/171) with subtype C and 1.2% (2/171) with recombinant forms (CRF_12_BF or CRF_02_AG).

**Figure 1:**
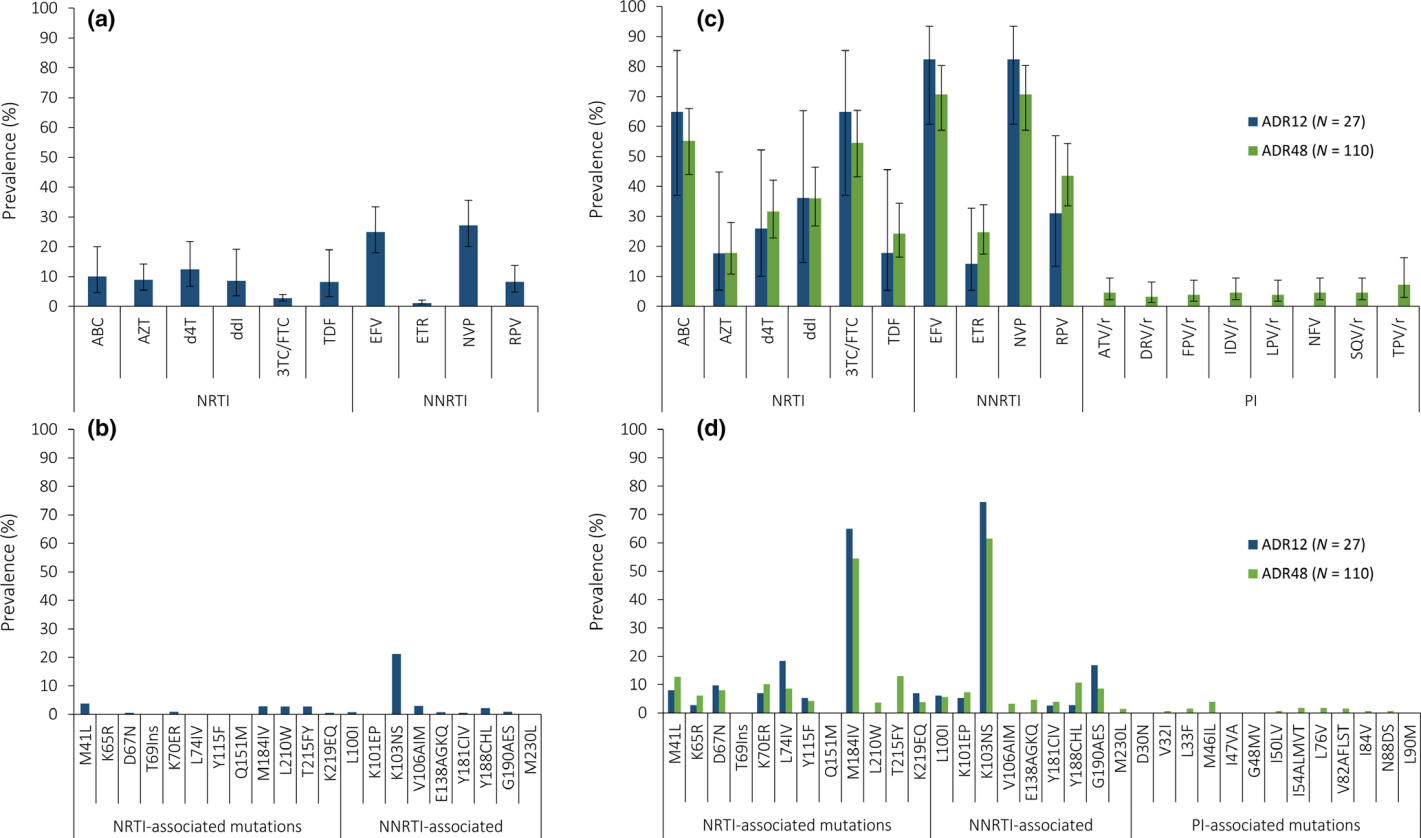
**Pretreatment and acquired HIV drug resistance prevalence and patterns in Nicaragua, 2016. Resistance was defined as a penalty score ≥15 using the Stanford HIVdb tool (v8.2).** **(a)** PDR prevalence by drug, **(b)** Frequency of PDR mutations, **(c)** ADR12/ADR48 prevalence by drug and **(d)** Frequency of ADR12/ADR48 mutations. Study design‐weighted proportions and 95% confidence intervals are shown. Only major HIVDR mutations are shown. No INSTI or PI mutations were observed for the PDR and ADR12 surveys. No INSTI mutations were observed for the ADR48 survey. 3TC/FTC, lamivudine/emtricitabine; ABC, abacavir; ADR12, acquired drug resistance at 12 (±3) months on ART; ADR48, acquired drug resistance at ≥48 months on ART; ATV/r, atazanavir/ritonavir; AZT, zidovudine; d4T, stavudine; ddI, didanosine; DRV/r, darunavir/ritonavir; EFV, efavirenz; ETR, etravirine; FPV/r, fosamprenavir/ritonavir; IDV/r, indinavir/ritonavir; INSTI, integrase strand transfer inhibitor; LPV/r, lopinavir/ritonavir; NFV, nelfinavir; NNRTI, non‐nucleoside reverse transcriptase inhibitor; NRTI, nucleoside reverse transcriptase inhibitor; NVP, nevirapine; PDR, pretreatment drug resistance; PI, protease inhibitor; RPV, rilpivirine; SQV/r, saquinavir/ritonavir; TDF, tenofovir; TPV/r, tipranavir.

The weighted national prevalence of PDR to any ARV was 23.4% (95% CI: 14.4% to 35.6%), and 19.3% (12.2% to 29.1%) to NNRTIs (Table 2). Nearly one in every five (19.3%, 12.2% to 29.1%) ART initiators had resistance to at least one of the drugs of the preferred first‐line ART regimen. As expected, NNRTI PDR was higher in ART initiators with prior ARV exposure compared with no exposure (76.2% vs. 11.0%, *p* < 0.001). Low‐levels of PDR were observed to TDF (2.9%, 0.8% to 10.4%) and FTC (3.5%, 1.1% to 10.2%), although these estimates had low precision (Figure 1a). Similarly, the study was not powered to observe differences in PDR prevalence by sex. No PDR was observed to PI or integrase strand transfer inhibitors (INSTI). The most frequent PDR mutation was K103NS (15.5%) (Figure 1b). No INSTI, or PI resistance‐associated mutations were observed.

**Table 2:** Prevalence of pretreatment and acquired HIV drug resistance in Nicaragua, 2016^a^

HIV drug resistance, n/N (%, 95% CI)	All	Male	Female
PDR			
All ART initiators			
Any	40/171 (23.4, 14.4 to 35.6)	22/123 (17.9, 9.9 to 30.2)	18/48 (37.5, 20.8 to 57.8)
NNRTI	33/171 (19.3, 12.2 to 29.1)	18/123 (14.6, 8.0 to 25.3)	15/48 (31.3, 19.6 to 45.8)
NRTI	18/171 (10.5, 4.9 to 21.1)	11/123 (8.9, 4.0 to 18.8)	7/48 (14.6, 5.0 to 35.5)
ART initiators without prior ARV exposure			
Any	23/146 (15.8, 8.8 to 26.6)	16/112 (14.2, 7.4 to 25.4)	7/34 (20.6, 7.5 to 45.6)
NNRTI	16/146 (11.0, 6.0 to 19.3)	12/112 (10.7, 5.2 to 20.6)	4/34 (11.8, 5.5 to 29.8)
NRTI	10/146 (6.8, 2.7 to 16.1)	7/112 (6.2, 23.2 to 15.5)	3/34 (8.8, 1.6 to 36.8)
ART initiators with prior ARV exposure			
Any	16/21 (76.2, 52.9 to 90.1)	6/8 (75.0, 22.9 to 96.8)	10/13 (76.9, 47.1 to 92.6)
NNRTI	16/21 (76.2, 52.9 to 90.1)	6/8 (75.0, 22.9 to 96.8)	10/13 (76.9, 47.1 to 92.6)
NRTI	7/21 (33.3, 13.9 to 60.8)	4/8 (50.0, 10.0 to 90.0)	3/13 (23.1, 5.9 to 58.8)
ADR12			
PLHIV on ART			
Any	22/114 (18.1, 10.6 to 29.2)	17/76 (15.6, 9.4 to 24.7)	5/38 (23.2, 7.5 to 53.0)
NNRTI	21/114 (17.5, 10.1 to 28.7)	16/76 (14.7, 9.0 to 23.2)	5/38 (23.2, 7.5 to 53.0)
NRTI	17/114 (14.9, 7.9 to 26.5)	13/76 (11.9, 6.8 to 20.0)	4/38 (21.0, 5.9 to 52.8)
NNRTI + NRTI	16/114 (14.4, 7.4 to 26.1)	12/76 (11.0, 6.3 to 18.6)	4/38 (21.0, 5.9 to 52.8)
PLHIV on ART with VL ≥1000 copies/mL			
Any	22/27 (85.1, 66.1 to 94.4)	17/18 (94.7, 64.9 to 99.4)	5/9 (75.0, 28.3 to 95.8)
NNRTI	21/27 (82.4, 60.7 to 93.4)	16/18 (89.4, 49.5 to 98.6)	5/9 (75.0, 28.3 to 95.8)
NRTI	17/27 (70.2, 43.6 to 87.8)	13/18 (72.4, 42.3 to 90.4)	4/9 (67.9, 18.7 to 95.1)
NNRTI + NRTI	16/27 (67.5, 40.0 to 86.6)	12/18 (67.1, 34.8 to 88.6)	4/9 (67.9, 18.7 to 95.1)
PLHIV on first‐line ART with VL ≥ 1000 copies/mL			
Any	22/27 (85.1, 66.1 to 94.4)	17/18 (94.7, 64.9 to 99.4)	5/9 (75.0, 28.3 to 95.8)
NNRTI	21/27 (82.4, 60.7 to 93.4)	16/18 (89.4, 49.5 to 98.6)	5/9 (75.0, 28.3 to 95.8)
NRTI	17/27 (70.2, 43.6 to 87.8)	13/18 (72.4, 42.3 to 90.4)	4/9 (67.9, 18.7 to 95.1)
NNRTI + NRTI	16/27 (67.5, 40.0 to 86.6)	12/18 (67.1, 34.8 to 88.6)	4/9 (67.9, 18.7 to 95.1)
PLHIV on first‐line NNRTI ART with VL ≥ 1000 copies/mL			
Any	21/25 (84.9, 65.4 to 94.4)	17/18 (94.7, 64.9 to 99.4)	4/7 (64.3, 18.1 to 93.6)
NNRTI	20/25 (81.3, 58.2 to 93.2)	16/18 (89.4, 49.5 to 98.6)	4/7 (64.3, 18.1 to 93.6)
NRTI	16/25 (65.3, 42.2 to 82.9)	13/18 (72.4, 42.3 to 90.4)	3/7 (50.3, 10.1 to 90.1)
NNRTI + NRTI	15/25 (61.7, 38.0 to 80.9)	12/18 (67.1, 34.8 to 88.6)	3/7 (50.3, 10.1 to 90.1)
ADR48			
PLHIV on ART			
Any	87/353 (22.2, 18.3 to 26.6)	58/219 (26.2, 20.9 to 32.3)	29/134 (16.9, 11.2 to 24.8)
NNRTI	82/353 (20.8, 17.2 to 24.9)	55/219 (25.1, 19.9 to 31.1)	27/134 (15.2, 9.9 to 22.5)
NRTI	68/353 (17.5, 14.2 to 21.2)	48/219 (21.4, 16.5 to 27.2)	20/134 (12.3, 7.6 to 19.3)
PI	6/353 (1.4, 0.8 to 2.3)	5/219 (1.9, 1 to 3.6)	1/134 (0.6, 0.1 to 4.0)
NNRTI + NRTI	63/353 (16.1, 13.1 to 19.6)	45/219 (20.3, 15.5 to 26.1)	18/134 (10.5, 6.4 to 16.9)
PLHIV on ART with VL ≥1000 copies/mL			
Any	87/110 (75.5, 63.5 to 84.5)	58/71 (82.9, 72 to 90.2)	29/39 (63.8, 39.3 to 82.8)
NNRTI	82/110 (70.7, 58.7 to 80.3)	55/71 (79.4, 68 to 87.4)	27/39 (57.2, 34.5 to 77.2)
NRTI	68/110 (59.4, 48.1 to 69.8)	48/71 (67.7, 53.9 to 79.0)	20/39 (46.4, 27.5 to 66.4)
PI	6/110 (4.6, 2.2 to 9.5)	5/71 (6.1, 2.7 to 13.4)	1/39 (2.2, 0.2 to 19.8)
NNRTI + NRTI	63/110 (54.6, 43.3 to 65.4)	45/71 (64.2, 50.3 to 76.0)	18/39 (39.7, 22.6 to 59.9)
PLHIV on first‐line ART with VL ≥1000 copies/mL			
Any	75/93 (77.3, 63.3 to 87.0)	49/58 (85.6, 73.2 to 92.8)	26/35 (64.2, 37 to 84.6)
NRTI	70/93 (71.6, 57.7 to 82.3)	46/58 (81.4, 68.4 to 89.8)	24/35 (56.2, 31.3 to 78.4)
NNRTI	58/93 (59.7, 46.8 to 71.4)	41/58 (70.2, 54.5 to 82.3)	17/35 (43.2, 23.5 to 65.4)
PI	2/93 (1.8, 0.5 to 6.0)	2/58 (2.9, 0.7 to 10.7)	0/35
NNRTI + NRTI	53/93 (54, 41.3 to 66.2)	38/58 (66.0, 50.4 to 78.7)	15/35 (35.2, 18 to 57.4)
PLHIV on first‐line NNRTI ART with VL ≥1000 copies/mL			
Any	69/80 (82.4, 66.4 to 91.8)	46/51 (91.9, 80.6 to 96.9)	23/29 (68.1, 36.7 to 88.7)
NNRTI	66/80 (78.1, 62.3 to 88.4)	45/51 (90.4, 78 to 96.2)	21/29 (59.4, 31.3 to 82.5)
NRTI	53/80 (63.8, 49.5 to 76.0)	38/51 (74.5, 56.7 to 86.7)	15/29 (47.6, 25.3 to 71.0)
PI	2/80 (1.8, 0.4 to 7.3)	2/51 (3.1, 0.7 to 12.1)	0/29
NNRTI + NRTI	50/80 (59.4, 45.3 to 72.2)	37/51 (73, 54.9 to 85.7)	13/29 (39.0, 19.3 to 63.0)

ART, antiretroviral therapy; ARV, antiretroviral; ATV/r, atazanavir/ritonavir; CI, confidence interval; DRV/r, darunavir/ritonavir; EFV, efavirenz; FTC, emtricitabine; HIVDR, HIV drug resistance; LPV/r, lopinavir/ritonavir; NNRTI, non‐nucleoside reverse transcriptase inhibitors; NRTI, nucleoside reverse transcriptase inhibitors; NVP, nevirapine; TDF, tenofovir.

a Study design‐weighted proportions and 95% confidence intervals. Resistance was defined as a penalty score ≥15 using the Stanford HIVdb tool (v8.2). Any HIVDR was defined as resistance to one or more of the following drugs: NVP, EFV, any NRTI, DRV/r, LPV/r, or ATV/r; HIVDR to NRTI was defined as resistance to any NRTI; HIVDR to NNRTI was defined as resistance to NVP and EFV. Preferred first‐line ART regimen: TDF + FTC+EFV.

Our study suggests high NNRTI PDR level in Nicaragua, crossing the 10% threshold defined by the WHO for urgent programmatic action 24. As previously observed in other countries 25, NNRTI PDR was higher in ART initiators with prior ARV exposure compared with persons with no exposure, indicating a risk group amenable to immediate intervention. Although the goal sample size for the survey was not achieved, a critical limitation that may affect representativeness and produce estimates with wider CIs, we accounted for this limitation by applying a weighting process to estimate survey outcomes. Using this approach, the lower end of the 95% CI for NNRTI PDR was 12.2%, which provides strong evidence that NNRTI PDR has crossed the 10% threshold in Nicaragua.

Given the observed results, the National HIV Program might consider initiating ART with non‐NNRTI‐based regimens as the preferred option, prioritizing ART initiators with prior exposure to ARVs 24, 26. The observed low‐level rates of PDR to TDF and FTC suggests that an ART backbone based on these drugs could be effective. Considering this scenario, the WHO‐recommended fixed‐dose combination of TDF, 3TC and dolutegravir may be a good option for the preferred first‐line ART regimen in Nicaragua 27.

### 3.3 Acquired HIV drug resistance surveys

Several obstacles were met to reach the goal sample size for the ADR12 survey. The national retention rate at 12 months after ART initiation for adults ≥15 years old in Nicaragua was low (72%) for the survey period 21, representing a considerable challenge for survey enrolment. We attempted to account for this limitation by weighting the survey outcomes by eligible population size per clinic, number of eligible individuals enrolled and proportion of successful sequences, as well as adjusting for the national ART retention rate. Considering this limitation, the weighted national estimate prevalence of VL suppression was 77.8% (95% CI: 67.1% to 85.8%) among PLHIV on ART for 12 (±3) and 70.3% (66.7% to 73.8%) among those on ART for ≥48 months (Table 3). The retention‐adjusted VL suppression estimate was 56.0% (48.1% to 63.9%) among PLHIV on ART for 12 (±3) months.

**Table 3:** Prevalence estimates of viral load suppression among PLHIV on ART for 12 (±3) or ≥48 months, Nicaragua, 2016^a^

	ADR12		ADR48	
	n/N	% (95% CI)	n/N	% (95% CI)
PLHIV on ART	86/114	77.8 (67.1 to 85.8)	240/353	70.3 (66.7 to 73.8)
Male	58/76	83.5 (73.8 to 90.2)	148/219	68.7 (62.2 to 74.5)
Female	28/38	66.4 (42.7 to 84.0)	92/134	72.5 (65.2 to 78.8)
≤25 years	21/27	77.2 (45.6 to 93.2)	11/27	51.5 (23.0 to 79.1)
>25 years	65/87	78.2 (70.9 to 84.0)	229/326	72.2 (68.1 to 75.9)
PLHIV on first‐line ART	82/110	77.3 (66.4 to 85.5)	208/303	70.9 (66.8 to 74.6)
Male	54/72	83.0 (79.0 to 86.3)	126/184	69.2 (63.5 to 74.3)
Female	28/38	66.4 (45.0 to 82.7)	82/119	73.2 (66.9 to 78.7)
≤25 years	21/27	77.2 (44.7 to 93.4)	8/21	55.9 (37.7 to 72.6)
>25 years	61/83	77.4 (70.9 to 82.8)	200/282	72.3 (68.0 to 76.2)
PLHIV on first‐line NNRTI‐based ART	78/104	80.7 (76.1 to 84.7)	182/264	70.3 (65.7 to 74.4)
Male	54/72	83.0 (79.0 to 86.3)	112/163	68.9 (62.8 to 74.4)
Female	24/32	74.7 (60.5 to 85.1)	70/101	72.1 (64.7 to 78.4)
≤25 years	21/26	88.9 (81.2 to 93.7)	5/16	33.3 (14.5 to 59.5)
>25 years	57/78	76.8 (69.7 to 82.6)	177/248	72.9 (68.2 to 77.1)

ADR12, acquired drug resistance at 12 (±3) months on ART; ADR48, acquired drug resistance at ≥48 months on ART; ART, antiretroviral therapy; CI, confidence interval; NA, not applicable; NNRTI, non‐nucleoside reverse transcriptase inhibitor; PLHIV, people living with HIV.

a Study design‐weighted proportions and 95% confidence intervals. VL suppression was defined as <1000 copies/mL.

The prevalence of VL suppression in Nicaragua was lower than that observed in a systematic review of ADR studies in adults from LMICs during the period 2014 to 2017 28. High PDR prevalence may partially explain suboptimal VL suppression levels at 12 (±3) months on ART. However, the even lower rate of VL suppression observed among persons on ART for ≥48 months suggests programmatic weaknesses that need to be addressed. No differences on VL suppression associated to sex or age were evident, although the low precision of estimates may reduce study power to detect differences. Analysis of WHO early warning indicators of HIVDR and the identification of specific ART sites with poor performance could be useful to ascertain intervention opportunities 29, such as: adherence strengthening programmes, loss‐to‐follow‐up prevention, improvement of VL testing coverage and prevention of drug stock‐outs 8.

The prevalence of HIVDR among PLHIV on ART who had not achieved VL suppression, was 85.1% (95% CI: 66.1% to 94.4%) in ADR12 and 75.5% (63.5% to 84.5%) in ADR48 (Table 2). PI resistance was only observed in the ADR48 survey, 4.6% (2.2% to 9.5%) among those with unsuppressed VL. No INSTI resistance was observed in any of the surveys. HIVDR to EFV or NVP was >70% among PLHIV with unsuppressed VL in both ADR surveys (Figure 1c). The proportion of HIVDR to at least one NRTI of the preferred second‐line regimen, among PLHIV on first‐line ART with unsuppressed VL, was 64.9% in ADR12 and 54.5% in ADR48. The most frequent mutations were K103NS (ADR12: 74.4%; ADR48: 61.5%) and M184IV (ADR12: 64.9%; ADR48: 54.5%) (Figure 1d).

The high levels of HIVDR to NNRTI and NRTI among individuals on ART with unsuppressed VL are consistent with previously observed data in LMICs 28. The high proportion of HIVDR to NRTI suggests prolonged periods of exposure to suboptimal ART, warranting actions for rapid identification and switch of PLHIV failing first‐line ART, in order to preserve the NRTI backbone. Overall, ADR mutation patterns were similar to those observed in other Central American countries, with predominance of K103NS and M184V 30, 31. Our results suggest that currently recommended second‐line PI‐based regimens in Nicaragua remain an effective option for most individuals failing first‐line 3.

Approximately 20% of PLHIV receiving first‐line ART with VL ≥1000 copies/mL in Nicaragua did not have HIVDR. As previously shown 32, enhanced and tailored adherence support programmes, associated with a strengthened VL testing system, could be useful for such individuals to achieve viral suppression without the need to switch to a second‐line ART regimen.

## 4 Conclusions

Despite implementation challenges possibly affecting national representativeness and yielding low‐precision HIVDR estimates, important aspects of HIVDR were observed, including a high NNRTI PDR prevalence, above the 10% threshold suggested by the WHO for urgent programmatic action. This statistic combined with low viral suppression rates and high ADR levels warrant programmatic action. Negotiations to introduce non‐NNRTI‐based first‐line ART regimens and the strengthening of VL monitoring and follow‐up activities would benefit the national HIV programme.

We acknowledge that the surveys were implemented in 2016, which implies that HIVDR estimates may have changed with time. However, looking at increasing global and regional NNRTI PDR trends 1, 25, our data suggest that HIVDR could become an even more relevant issue in Nicaragua.

## Competing interests

The authors do not have any commercial or other associations that might pose a conflict of interest.

## Authors’ contributions

A.G.C., R.M.B., M.R., S.I.J., G.R., S.N., S.A.R. and G.R.T. designed the research study. C.G.M., D.T.T., M.P.G. and V.S.Q.M. performed HIV sequencing. A.G.C., R.M.B. and M.R. coordinated participant enrolment and data capture. C.V., R.G. and L.R., supervised implementation of field activities. A.S. and E.S. contributed with data management. A.G.C., R.M.B. and C.G.M. analysed the data. A.G.C., R.M.B. and S.A.R. wrote the manuscript. All authors have reviewed and approved the final manuscript.
